# Lombard effect, intelligibility, ambient noise, and willingness to spend time and money in a restaurant amongst older adults

**DOI:** 10.1038/s41598-022-10414-6

**Published:** 2022-04-21

**Authors:** Pasquale Bottalico, Rachael N. Piper, Brianna Legner

**Affiliations:** Department of Speech and Hearing Science, University of Illinois, 901 S. 6th St., Urbana-Champaign, IL 61820 USA

**Keywords:** Psychology and behaviour, Socioeconomic scenarios, Risk factors

## Abstract

Dining establishments are an essential part of the social experience. However, they are often characterized by high levels of background noise, which represents a barrier to effective communication. This particularly affects people suffering from hearing problems. Moreover, noise level exceeding normal conversational levels causes a phenomenon called the Lombard Effect, an involuntary tendency to increase the amount of vocal effort when talking in the presence of noise. Adults over 60 years represent the second largest population in the US and the majority of them suffer from some degree of hearing loss. The primary aim of the current study was to understand the effect of noise on vocal effort and speech intelligibility in a restaurant setting for adults over 60 years old with and without hearing loss. The secondary aim was to evaluate their perception of disturbance in communication and their willingness to spend time and money in a restaurant was affected by the varying levels of background noise. The results of this study showed background noise levels lower than 50 dB(A) will allow senior customers to minimize their vocal effort and to maximize their understanding of conversations, even for those with moderate to severe hearing loss. By setting a limit, it will also keep perceived disturbance low and willingness to spend time and money high among dining patrons.

## Introduction

Beyond the basic purpose of restaurants to provide food and drink, restaurants have, historically, fulfilled a human need for connection and shaped social relations. However, restaurants are often characterized by high level of background noise that negatively affect the communication.

Before the 1990’s, “excessive” restaurant noise was not negatively perceived by diners and critics because it was supposed to add to the atmosphere of the eating establishment^1^. A restaurant’s atmosphere is vital to keeping and enticing new patrons, which can cause the diners to increase their overall bill at a restaurant^1,2^. However, depending on the age group, several individuals might recall the noisy environment more than the food^3^. This is especially problematic for patrons aged 60 and older suffering from hearing loss, as background noise interferes with the comprehension of conversations^4–6^.

In recent years, there has been increased awareness towards the acoustics of eating establishments with the goal of improving speech intelligibility^6,7^. Restaurants are designed to look aesthetically pleasing and modernized, which often is portrayed with a lack of materials used to absorb sound. The lack of these materials tends to increase the overall noise level in the restaurant which has caused diners, critics, audiologists and researchers to take notice^1,3,5–12^. In 2018, a repeated restaurant survey revealed noise was the primary complaint of restaurant patrons^12^. Restaurants have a wide range of noise during hours of operation, which has caused restaurant critics to carry sound level meters to evaluate the noise levels to include in their reports^1^. Typical restaurant noise can fluctuate throughout the course of an evening ranging from 65 dB(A) up to as high as 85 dB(A)^1,3,4,7,13^. This causes increased difficulty to be heard and understood by anyone^4,5,10,13^. The main source of noise in a restaurant is caused by the patrons, as a consequence several researches suggested that dining establishments should no longer be thought to have “maximum capacity” but rather “acoustical capacity”^5,6,10^. The maximum capacity can be defined as the number of seats occupied within the restaurant space, in order to retain efficiency and safety to both the workers and patrons. The acoustical capacity is instead defined as “the maximum number of persons in a room for sufficient quality of verbal communication;” it can be calculated using the volume of the room (m) and the reverberation time (s) of the unoccupied, furnished restaurant setting^6^. It is possible to maximize the acoustical capacity by making design adjustments like carpeting, sound absorbent panels, or curtains on the walls and ceilings^1,6,8,9,14,15^.

The presence of high level background noise in an environment, where communication is key, often triggers the Lombard Effect. The Lombard Effect is the involuntary tendency of the speaker to increase the vocal effort, while speaking in loud noise^16–18^. The typical slope for the Lombard Effect is 0.3–0.6 dB in voice increase per dB in noise increase, when the noise exceeds 50 dB(A)^19,20^. In a recent study, Bottalico^21^ investigated the Lombard Effect values in restaurant settings, using typical restaurant noise in a laboratory. During the experiment, the author presented recorded restaurant noise at varying sound pressure levels from 35 dB(A) to 85 dB(A) with 5 dB increments to normal hearing college students. After reading a short passage (The Rainbow Passage)^22^, in each noise condition, participants were asked to score their perceived disturbance, how much time they would stay, and how much money they would be willing to spend. The results indicated a change-point of the Lombard Effect at 57.3 dB(A), with a slope of the lower segment being 0.31 dB/dB and the upper segment as being 0.54 dB/dB slope. Additionally, the college students’ scores for disturbance, time, and money started to decrease at 52.5 dB(A) (disturbance=52.2 dB(A), willingness=51.3 dB(A), money=52.5 dB(A)). The results form Bottalico^21^ had some limitations: (1) college students do not spend much dining out potentially due to lower income^23,24^, and (2) younger adults have a greater tolerance to loud sounds^25^. For this reason, this study will focus on adults older than 65 years.

Over the last hundred years, life expectancy has increased upwards of thirty years^26,27^; and by 2030, 19.3% of the population will be adults aged 65 years and older in the United States^28^. The National Institute on Aging (NIA) estimates there are approximately 49 million Americans aged 65 and older, which is the largest number of older adults than any other time recorded in history^29^. Recently in research, there has been an increased demand to better understand the process of aging to assist this population, not just in terms of healthcare^29^, but also to keep them connected to society^27^.

This population of older adults (aged 65+) has been shown to have a lower tolerance to loud sound versus their younger counterparts. This could be contributing to the cause of why older adults are choosing to avoid dining out. Another reason could be that there are several comorbidities, such as high-frequency hearing loss^30,31^ and voice disorders^32^, which are prevalent amongst older adults. The World Health Organization^33^ estimates approximately 60% of adults aged 60 and older will experience an age-related hearing loss at a degree of moderate loss or greater. It is possible the combination of high noise levels in the restaurant, in conjunction with hearing problems, makes conversing difficult for them to partake in and comprehend^4–6,31,34^.

With the goal of understanding how the senior population will react to noise in a restaurant setting, in terms of voice production (the Lombard Effect) and speech perception (the Cocktail Party Effect), the following research will investigate five different outcomes. The aim of this study will attempt to answer the following research questions: (1) What is the level of noise at which older adults experience the Lombard Effect? (2) What is the relationship perceived amount of disturbance and noise level in a restaurant for older adults? (3) How much time would older adults be willing to stay in restaurants with different noise levels? (4) How much money would they be willing to spend? (5) What is the noise level at which older adults are no longer able to have an intelligible conversation?

The hypotheses of the current study are: (1) Older adults will have a lower Lombard Effect than the previous young adult group, as a result of aging and decreased vocal effort^32^. (2) Older adults will perceive a greater disturbance than the young adult group, due to younger adults’ lesser likelihood of peripheral hearing loss and more reliable auditory processing abilities^5,31,35^. 3) Older adults’ willingness to spend time and money will decrease as the noise increases, due to their dislike of louder noise levels while dining out^5^. 4) Older adults will perform poorer on intelligibility as the noise increases, due to their recorded peripheral hearing loss, lack of amplification to assist in preservation of word understanding abilities, and their general decrease in cognition specifically for selective attention, working memory, and auditory closure abilities. 5) The degree of hearing loss will amplify the aforementioned effects.

## Results

**Figure 1:**
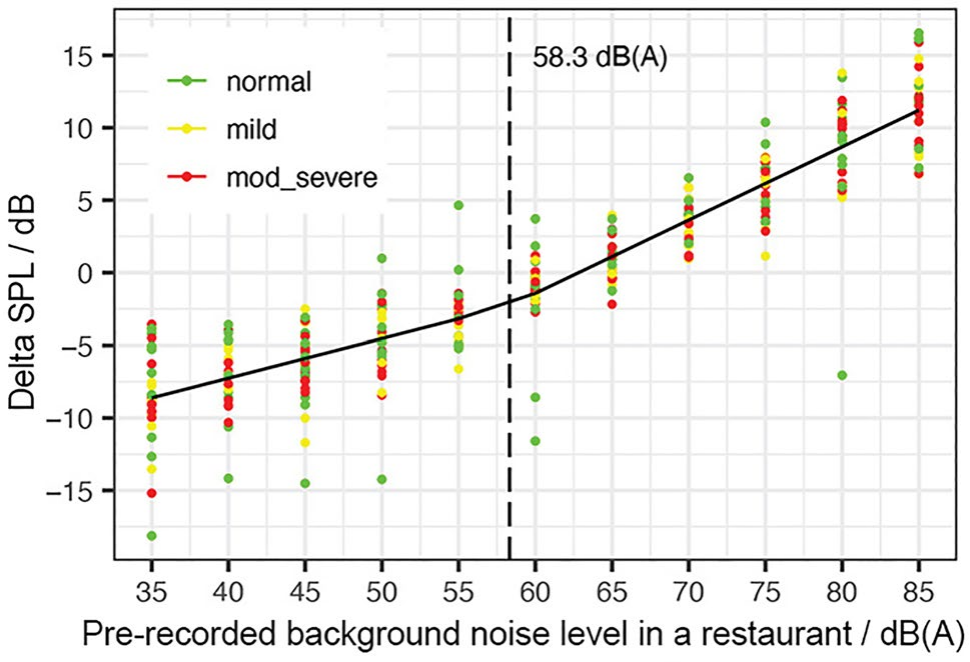
Relationship between the pre-recorded background noise level in a restaurant in dB(A) and Delta SPL in dB. Vertical dashed line marks the change-point.

A piecewise linear (also called segmented or broken-line) models appear to be the best fits in comparison to simple linear models and quadratic models. The goodness of the fit was evaluated based on the R-squared, the analysis of the residuals, and the fact that the use of a more complex model did not improve the fit in a statistically significant way. The Delta SPL was measured at each of the 11 noise levels between 35 and 85 dB(A), as shown in Fig. 1. A piecewise linear model was fit to the response variable, Delta SPL, and the predictor, Ln. The presence of Hearing Loss (HL) did not statistically influence the Lombard Effect. The piecewise linear model individuates a breakpoint in Ln at 58.3 dB(A) (CI 95% lower: 53.4, CI % upper: 63.3). The slope of the lower segment was 0.27, and the upper was 0.51, with an R-squared of 0.86. Model estimates with associated standard errors and *p* values are given in Table 1.

**Figure 2:**
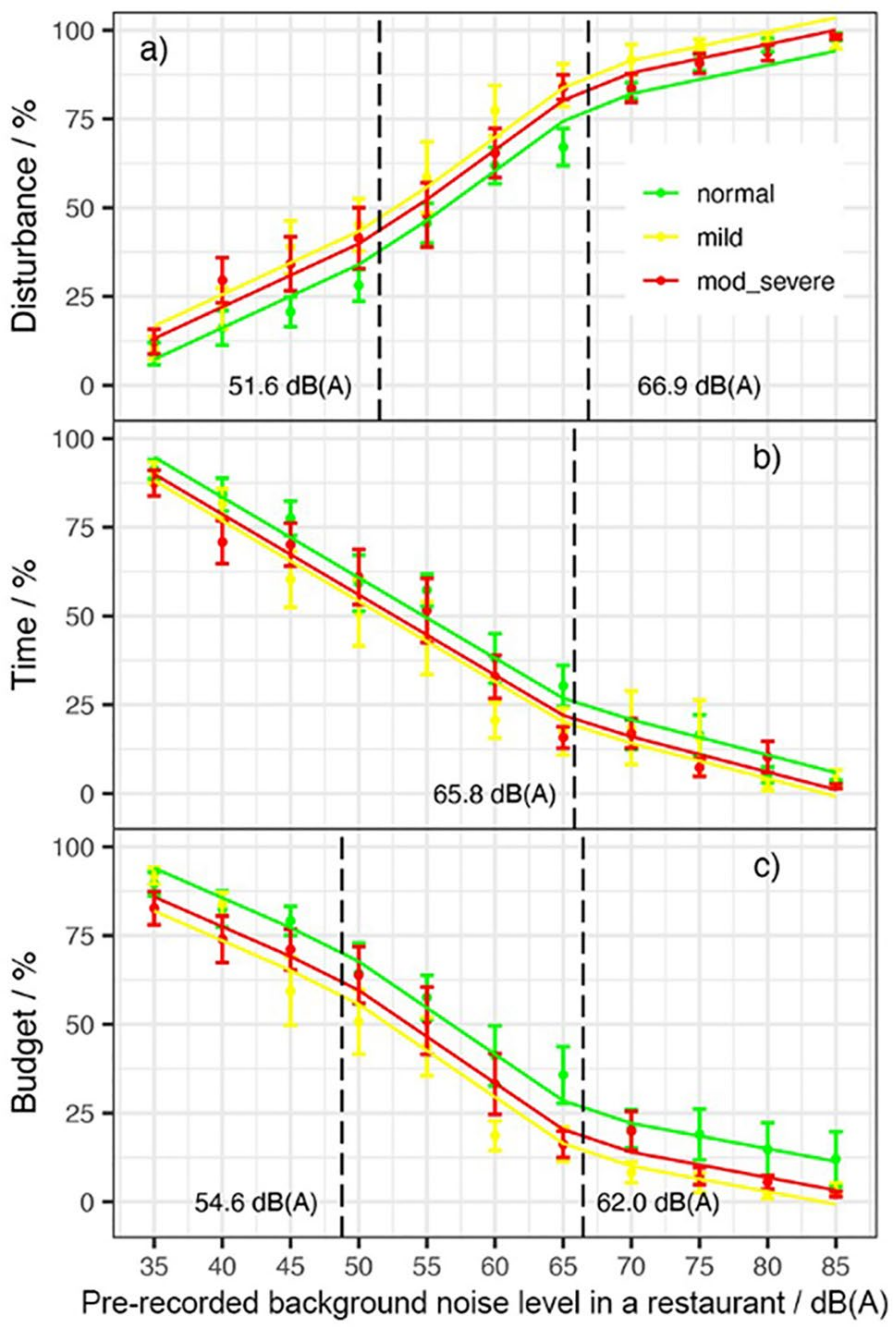
Relationship between the pre-recorded background noise level in a restaurant in dB(A) and self-reported communication disturbance (**a**), willingness to spend time (**b**), and willingness to spend money (**c**), where the error bands indicate the standard error. Vertical dashed lines mark the change-points.

Self-reported communication disturbance was measured at each of the 11 noise levels (Fig. 2, panel a). A piecewise linear model was fit to the response variable, disturbance (% of “very high”) and the predictors, Ln and HL. The piecewise linear model individuates two breakpoints, the first at 51.6 dB(A) (CI 41.4–60.3) and the second at 66.9 dB(A) (CI 62.0–71.2). The slope of the lower segment was 1.77 percent-points per dB (pp/dB), the medium was 2.78 (pp/dBA) and the upper was 0.83 (pp/dBA). In the same noise level condition, compared to participants with normal hearing, participants with mild HL scored the disturbance 9.37% higher, while participants with moderate to severe HL scored the disturbance 5.87% higher. The R-squared of the model was 0.78. Model estimates with associated standard errors and *p* values are given in Table 1.

The willingness to spend time in the restaurant was measured at each of the 11 noise levels, as shown in Fig. 2 (panel b). A piecewise linear model was fit to the response variable, time willing to stay (% of “A long time”) and the predictors, Ln and HL. The piecewise linear model individuates a breakpoint in Ln at 65.8 dB(A) (CI 58.9–72.7). The slope of the lower segment was − 2.27 (pp/dBA), and the upper was − 1.00 (pp/dBA). In the same noise level condition, compared to participants with normal hearing, participants with mild HL scored the time willing to stay 6.62% lower, while participants with moderate to severe HL scored the disturbance 4.79% lower. The R-squared of the model was 0.73. Model estimates with associated standard errors and *p* values are given in Table 1.

The willingness to spend money in the restaurant was measured at each of the 11 noise levels, as shown in Fig. 2 (panel c). A piecewise linear model was fit to the response variable, money willing to spend (% of “All the budget”) and the predictors, Ln and HL. The piecewise linear model individuates two breakpoints, the first at 54.6 dB(A) (CI 40.9–60.0) and the second at 62.0 dB(A) (CI 58.0–66.0). The slope of the lower segment was − 1.80 percent-points per dB (pp/dB), the medium was − 3.86 (pp/dBA) and the upper was − 0.87 (pp/dBA). In the same noise level condition, participants with mild HL scored the time willing to stay 12.06% lower compared to participants with normal hearing, while participants with moderate to severe HL scored the disturbance 8.11% lower compared to participants with normal hearing. The R-squared of the model was 0.69. Model estimates with associated standard errors and *p* values are given in Table 1.

**Table 1:** Piecewise Linear model output for four models with response variables DeltaSPL, Disturbance, Time and Budget as a function of Ln and Hearing Loss (with reference level equal to Normal Hearing).

Predictor	Estimate	st.error	t-value	*p* value	Ln range dB(A)
DeltaSPL / dB(A)					
(Int.)	− 18.07	1.39	− 13.01	< 0.001***	
HL Mild	− 0.12	0.37	− 0.31	0.76	
HL Mod-Sev	− 0.03	0.34	− 0.10	0.92	
Ln	0.27	0.03	9.01	< 0.001***	[35–58.4]
Ln	0.51	0.04	6.19	< 0.001***	[58.5–85]
Disturbance / %					
(Int.)	− 54.75	11.31	− 4.84	< 0.001***	
HL Mild	9.37	2.25	4.16	< 0.001***	
HL Mod-Sev	5.87	2.07	2.84	0.005**	
Ln	1.77	0.26	6.76	< 0.001***	[35–51.6]
Ln	2.78	0.49	2.06	0.04*	[51.7–66.9]
Ln	0.83	0.49	− 4.03	< 0.001***	[67–85]
Time / %					
(Int.)	174.05	6.43	27.05	< 0.001***	
HL Mild	− 6.63	2.51	− 2.64	0.008**	
HL Mod-Sev	− 4.79	2.30	− 2.08	0.038*	
Ln	− 2.27	0.12	− 18.33	< 0.001***	[35–65.8]
Ln	− 1.00	0.32	4.00	< 0.001***	[65.9–85]
Budget / %					
(Int.)	157.30	13.57	11.59	< 0.001***	
HL Mild	− 12.06	2.70	− 4.46	< 0.001***	
HL Mod-Sev	− 8.11	2.48	− 3.27	0.002**	
Ln	− 1.80	0.31	− 5.71	< 0.001***	[35–54.5]
Ln	− 3.92	1.04	− 2.03	0.043*	[54.6–62.1]
Ln	− 0.87	1.02	2.97	0.003**	[62.2–85]
Intelligibility Scores / %					
(Int.)	134.35	10.14	13.25	< 0.001***	
HL Mild	− 15.66	7.38	− 2.12	0.03*	
HL Mod-Sev	− 65.61	6.77	− 9.69	< 0.001***	
Ln	− 1.08	0.23	− 4.73	< 0.001***	[35–51.2]
Ln	− 3.39	0.31	− 7.47	< 0.001***	[51.3–71.3]
Ln	− 0.65	0.41	6.68	< 0.001***	[71.4–85]
Ln:HL Mild	0.10	0.12	0.80	0.423	
Ln:Mod-Sev	0.76	0.11	6.92	< 0.001***	

Significance codes: ‘***’<0.001; ‘**’<0.01; ‘*’ <0.05.

**Figure 3:**
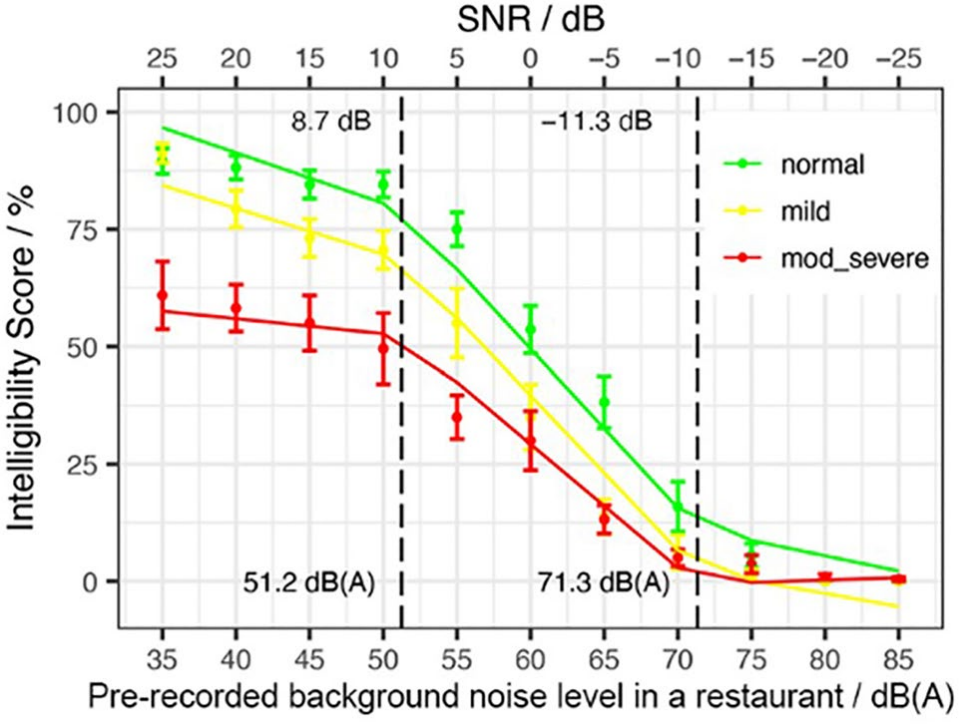
Intelligibility scores as function of SNR dB (labels on the top) and level of the pre-recorded background noise level in a restaurant in dB(A) (labels on the bottom). Vertical dashed lines mark the change-points.

The intelligibility in the restaurant was measured at each of the 11 noise levels, as shown in Figure 3. A piecewise linear model was fit to the response variable, intelligibility scores (% of correct word recognized) and as predictors, Ln, HL and their interaction. The piecewise linear model individuates two breakpoints, the first at 51.2 dB(A) (CI 48.2–54.2) and the second at 71.3 dB(A) (CI 68.4–74.2). Because of the interaction between Ln and HL, in each of the three segment there is a slope for each HL levels (Normal, Mild and Moderate-Severe). The slopes of the lower segments were − 1.08, − 0.98, and − 0.32 percent-points per dB (pp/dB) for participants with Normal Hearing, Mild HL and Moderate-Severe HL, respectively. The slopes of the medium segments for the three groups were − 3.39, − 3.30, and − 2.64 percent-points per dB (pp/dB), while the slopes of the upper segments for the three groups were − 0.65, − 0.56, and 0.10 percent-points per dB (pp/dB). The R-squared of the model was 0.85. Model estimates with associated standard errors and *p* values are given in Table 1.

## Discussion

The aim of this study was to identify which levels of background noise are affecting negatively the dining experience for adults over 60 years with and without hearing loss. The parameters analyzed were objective, i.e. Lombard Effect and speech intelligibility, and subjective, i.e. the perceived disturbance, the willingness to spend time and the willingness to spend money. The long-term goal of this project is to increase noise awareness in crucial environments for social life, such as restaurants, in order to create age-friendly communities.

Regarding the relationship between noise and voice level (the Lombard Effect), the hypothesis that “older adults will have a lower Lombard Effect than college students”, was verified. Similarly, to the results of Bottalico^21^, there was no definitive starting point for the Lombard Effect in older adults, and a similar change-point was found at a noise level of about 57–58 dB(A). This noise level threshold is in agreement to previous reports of the Lombard Effect’s starting point occurring between 40–60 dB(A)^19,20^. College students and older adults showed a similar trend of increased vocal effort as the noise levels increased from 35 dB(A) to 85 dB(A). The Lombard Effect for older adults was slightly lower than the one for college students (0.51 dB/dBA and 0.54 dB/dBA, respectively); this is supporting the hypothesis in which vocal fatigue and aging influence the overall vocal effort of older adults^32^. The vocal behavior of the participants was not statistically different in the three different groups of hearing loss, which can be explained by previous research about the effect on hearing loss on voice production. Coelho *et al.*^36^ found that individuals with mild and moderate hearing loss can only present problems with resonances of the vocal tract, while severely impaired individuals may lack intensity and frequency control. Because among our participants only two had a severe hearing loss, it was predictable that the voice loudness would have not change among the three groups.

The hypotheses concerning disturbance, time, and money were confirmed: (1) the amount of older adults’ disturbance increased with the noise and (2) the willingness to spend time and money decreased as the noise increased. The communication disturbance perceived by the participants increased with noise starting from the lowest level of noise. At 51.6 dB(A), the slope of this relationship increased dramatically till a saturation point for the disturbance at 66.9 dB(A).

The relationship of the willingness to spend time in the restaurant with the background noise level in the restaurant showed one breakpoint at 65.8 dB(A). A saturation effect for noise levels higher than the breakpoint was present in the relationship. It appears that since from low background noise levels, participants were annoyed by it. However, once the noise was exceeding the breakpoint, participants reported that they would have left the dining facility as quickly as they had entered.

The relationship of the willingness to spend money in the restaurant with the background noise level in the restaurant showed two breakpoints with the lower segment ending at 54.6 dB(A) and an upper segment starting at 62.0 dB(A). Between those two levels, the willingness to spend money in the restaurant decreased quickly with a slope of − 4% of the budget per dB(A) in noise increase. Once the noise was exceeding 62.0 dB(A) participants reported that they would have spent less than 25% of their budget.

The results in terms of communication disturbance, willingness to spend time and willingness to spend money were significantly different if the participants were suffering from hearing loss (both mild and moderate-to-severe). Participants with normal hearing were better in tolerating the background noise levels. On equal noise conditions, they were less disturbed and willing to spend more time and money in that restaurant compared to participants with hearing loss. The present results also differ from the results of Bottalico^21^ where college students’ judgments were minimally impacted by noise levels lower than 50−55 dB(A). These differences between older and younger adults could be explained by their higher sensitivity to louder noise levels of older adults compared to the sensitivity of younger adults^25^.

Noise significantly influenced intelligibility and the following hypotheses were confirmed: (1) older adults performed worse on intelligibility as the noise increased and (2) the higher the degree of hearing loss the lower their overall intelligibility performance. Figure 3 showed a distinct difference amongst all hearing categories. Each hearing loss group begins at a different percentage of intelligibility in the first noise condition (Ln = 35 dB); this finding is important because the moderate-to-severe group’s understanding abilities are already approximately 50% in the presence of minimal noise when a talker uses a normal vocal effort (60 dBA) at one meter’s distance. The reduced intelligibility can be explained by the degree of their hearing loss, no visual cues from facial expressions or lip-reading due to the HATS and lack of auditory stimulation of their peripheral loss with assistive hearing technology. The model also showed two breakpoints, with the first occurring at 51.2 dB(A) and the second at 71.3 dB(A), corresponding to 8.8 dB of SNR and − 11.3 dB SNR. The first breakpoint occurs rather early in the noise levels, with stabilized performances, followed by a rapid decline at which point all groups saturate at 71.3 dB(A) with little to no speech understanding left by the Ln = 70 dB(A) noise condition. These change-points are significant, because not only there is a difference of performance among the hearing categories at the initial starting level, but also each group understands 50% of the words correct at different noise levels (normal = 60 dB(A); mild = 55 dB(A); moderate-to-severe = 50 dB(A)). This difference of 5–10 dB(A) per group suggests that the older adults have a wide range of speech processing abilities in the presence of background noise, especially without the assistance of amplification or visual facial cues and expressions. In summary, it is imperative to decrease background noise in restaurants if we want to create age-friendly environment. In particular, noise levels lower than 50 dB(A) will have a minimal impact on the speech intelligibility and vocal effort of older adults. This noise limit could also have a positive impact on the revenue of the restaurants.

This study had several limitations. This study focused on the background noise in a restaurant, and crucial factors in the success of a restaurant such as the quality of food, the service, the interior design were not considered. Music and other conversations mix together to create a sense of privacy within one’s own table. However, when the noise is higher than the level needed to guaranty a certain level of privacy, it can cause a breakdown in communication. In a dining atmosphere, traditionally there are several other patrons enjoying the same atmosphere within relatively close distance of each other, but in this experimental setting, only one table was present in the laboratory. This means that the participants were not considering at all their privacy. A second limitation is that the HATS does not show any visual facial expressions or move its lips. Subconsciously, humans look at another talker’s lips which helps to enhance the auditory signal; however, for those with a hearing impairment could cause them to rely more on lip reading as a compensatory strategy. Moreover, the HATS speech level did not increase with the background noise level, as naturally would happen^37^. In the present study, the participants did not use hearing aids, even if few of them were using them in their daily life. This represents a limitation to the ecological validity of the study. However, we considered that there are an estimated 22.9 million older Americans with audiometric hearing loss who do not use hearing aids^38^. Finally, even if presented in previous papers, the subjective evaluations of the disturbance in communication and the willingness to spend time and money in a restaurant were not externally validated and they can all be representative of the degree of displeasure caused by the noise level.

## Methods

### Participants

With protocol approval of the University of Illinois Urbana-Champaign’s Office for the Protection of Research Subjects Review Board (IRB No. 19479), a total of 31 individuals were recruited for the study. The experiment was performed in accordance with the guidelines and regulations suggested by the University of Illinois Urbana-Champaign’s Office for the Protection of Research Subjects Review Board. Inclusion criteria required the participant to be over the age of sixty years and have normal hearing (<25 dB HL) or hearing loss (> 25 dB HL); additionally, participants must be proficient English speakers, since the intelligibility test will be given in English and non smokers. The participants signed an informed consent form, completed a demographics form, and underwent a hearing test prior to completion of the experiment. The hearing test included otoscopy, tympanometry, speech reception thresholds, and air and bone conduction audiometry. An initial exclusion criterion, in order to continue with the Lombard Effect part of the study, involved pure tone air conduction results showing asymmetries greater than 10 dB HL, at two or more consecutive frequencies, or an air-bone gap greater than 15 dB HL, without a previous medical consultation with an otolaryngologist. Among the 31 participants (14 males and 17 females), 10 were with Normal Hearing, 10 with Mild HL, and 11 with Moderate-Severe HL.

### Procedure

After participants confirmed their age, a hearing screening was performed, including reviewed their hearing history, otoscopy, tympanometry, air conduction pure-tone audiometry with insert phones from 250–8000 Hz (with inter-octaves as necessary), speech reception thresholds in quiet, and unmasked or masked bone conduction pure-tone thresholds. Supra-aural headphones were used to reconfirm pure tone air conduction gaps of 10 dB HL or greater between ears at two or more consecutive frequencies.

After completion of the hearing test, the individual continued with the second part of the experiment. This included in order the Lombard Effect testing, speech-in-noise intelligibility testing, and scored on a visual analogue scale their perceptual responses of (1) communication disturbance, (2) willingness to spend time, and (3) willingness to spend money in a restaurant. Participants completed all components of the study in one or two forty-five to sixty-minute sessions, resulting from scheduling conflicts and/or participant fatigue.

### Hearing loss categorization

**Figure 4:**
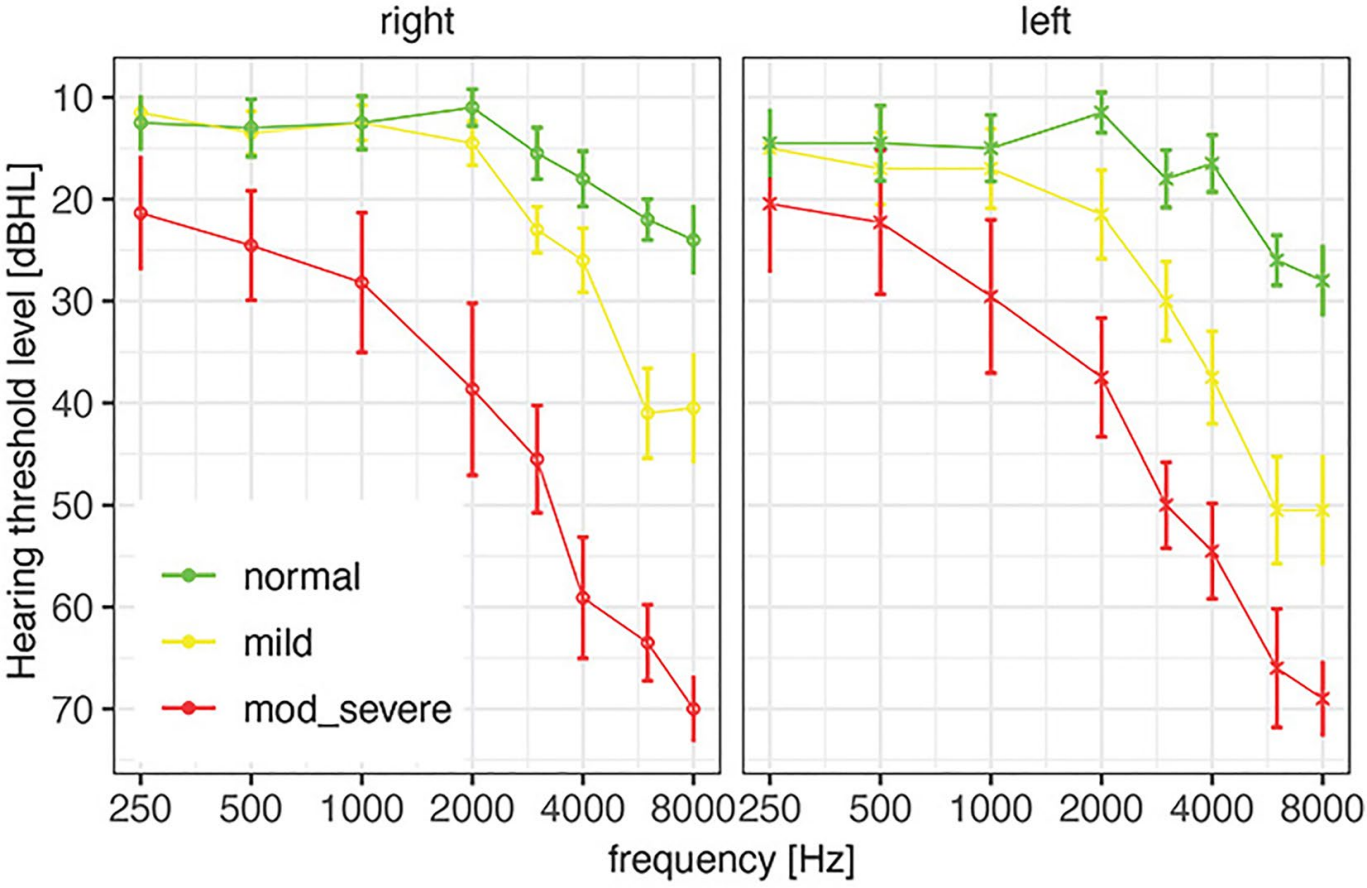
Average hearing thresholds per hearing loss category for the left and right ear.

After the hearing evaluation, participants were grouped in three categories. These categories were determined by the collective pure-tone averages of 2000 Hz, 4000 Hz, and 8000 Hz of both ears per participant. Average audiometric thresholds of the right and left ear per participant categories are represented in Figure 4. The calculated high frequency average was classified into: (1) normal hearing [<25 dB HL], (2) mild sensorineural hearing loss [26–40 dB HL], 3) moderate, severe, and profound sensorineural hearing loss [41+ dB HL]. The categorization was based on ASHA^39^ and Clark^40^ degrees of hearing loss.

### Measurements

**The Lombard Effect** Participants were fit with the head-mounted microphone connected to a portable recorder. Then, participants were asked to read in the presence of pre-recorded restaurant noise the first six sentences of “The Rainbow Passage”^22^, which is used by Speech Language Pathologists to assess and monitor voice and articulation disorders. The pre-recorded restaurant noise included patrons babble from a real restaurant and the clinking of dining ware and cutlery. The noise was presented at eleven levels from 35 dB(A) to 85 dB(A) with 5 dB interval in a random order. Participants were instructed, by the undergraduate student assistant, with the following: “Each time, I [the listener] would like you to pretend that we are talking in a restaurant and you are telling the story to me. Make sure that I understand you equally well each time.”

**Speech Intelligibility in Noise** The participants completed a speech-in-noise intelligibility test for each of the eleven, randomized noise conditions. Our intelligibility test was designed using the 250–300 standardized Northwestern University 6, NU-6^41^, words available to all audiologists. These words were recorded using the voice of an undergraduate student assistant; the words were individually clipped and then randomized into eleven, twenty-word lists. These recordings were then played through a Head and Torso Simulator (HATS) (GRAS, Holte, Denmark), placed at one-meter distance from the participant, to guarantee consistency in the speech material. The word lists were presented at a level of 60 dB(A), corresponding to a normal vocal effort at one meter of distance^42^. Participants were asked to repeat the word pronounced by the HATS. Intelligibility scores were measured as percentage of words correct.

**Disturbance, Time, and Budget Scores** Self-reported communication disturbance and willingness to spend time and money were measured on visual analog scales on paper. The participant was instructed to place a vertical tick on the horizontal line regarding their amount of disturbance, time, and money per noise condition. The paper document was then scanned and uploaded to be measured, in centimeters, using Adobe Acrobat Pro DC Software and the Adobe Software Measurement Tool (version 2022.001.20085, https://www.adobe.com/). The responses were then recorded in an Excel spreadsheet according to the participant’s identification number.

### Analysis of voice recordings

The audio recordings of participants were segmented using ELAN software (version 5.8, https://archive.mpi.nl/tla/elan)^43^. Each of the eleven “Rainbow Passage” segments were saved according to the participants identification number and noise level condition. MATLAB (R2017a) was used for speech signal analysis. In each condition, the equivalent SPL was measured. For each condition, the mean value of the SPL was obtained per subject. For each subject, the average of SPL among the conditions was computed and subtracted from each mean SPL values for that subject (termed Delta SPL). This within-subject centering was performed in order to evaluate the variation in the subject’s vocal behavior in the different noise conditions from their typical vocal behavior (mean value of the SPL per subject). Each participant was identified by their identification number, gender, age and type of hearing loss. As a first preliminary assessment of the reliability of the recordings, the Voice to Noise Ratio (VNR) was evaluated. To evaluate VNR, mixture of Gaussians models was used. In statistics, a mixture model is a probabilistic model for representing the presence of subpopulations within an overall population, without requiring that an observed data set should identify the sub-population to which an individual observation belongs. In our case, the time history of all the SPL recorded by the microphone represents the overall population. The two subpopulations are the Voice levels and the Noise levels. The distribution of the overall population was modeled as the sum of two Gaussian distributions, where two average values represented the Voice and the Noise average levels. The average VNR among the different noise conditions was 21.2 dB with a standard deviation of 2.0 dB. In the worst case (VNR = 19.3 dB), the contribution of the background noise on the overall level (noise and voice) was about 0.05 dB. This result confirms that the effect of noise on the equivalent level was acoustically negligible.

### Equipment and room measurements

The study was performed in a standardized sound-attenuated booth^44^. Reverberation time was measured in the sound booth from the impulse responses (IRs) generated by balloon pops^45^. The four IRs were recorded in two source positions and two microphone positions by means of an NTI Measurements microphone M2211 (Class 1 frequency response) and analyzed in one-third octave bands by means of an NTI XL2 Audio and Acoustic Analyzer. The reverberation time (T20) at mid-frequencies in the room was 0.05 s, while the background noise was 22.5 dB(A). The hearing test will be performed using a calibrated tympanometer (TympStar, Grason-Stadler, Eden Prarie, MN), audiometer (GSI 61, Grason-Stadler, Eden Prarie, MN), insert phones (3M E-A-RTONE 5A 410-5002), TDH-50 headphones, and bone oscillator. The participants’ speech will be recorded using a head-mounted microphone (Beta 54 WBH54, Shure, Niles, IL) and connected to an audio interface (US-, TASCAM [DR44], Montebello, CA). The same restaurant noise was emitted by two directional speakers (High performance 100 dB speaker, E3 Diagnostics, Arlington Heights, IL) placed at 45 to the sides of the participants.
